# Pen Yan Jing Tablets Alleviates Pelvic Inflammatory Disease by Inhibiting Akt/NF-κB Pathway

**DOI:** 10.7150/ijms.87433

**Published:** 2023-09-04

**Authors:** Ping Tang, Qi Ding, Juan Lin, Xinrong Yang, Yiting Wang, Fangle Liu, Yuying Zheng, Liuqing Lin, Deqin Wang, Baoqin Lin

**Affiliations:** 1 Experimental Center, The First Affiliated Hospital of Guangzhou University of Chinese Medicine, Guangzhou, 510405, China.; 2 Hutchison Whampoa Guangzhou Baiyunshan Chinese Medicine Co., Ltd., Guangzhou, 510515, China.; 3 School of Pharmaceutical Sciences, Guangzhou University of Chinese Medicine, Guangzhou, 510006, China.

**Keywords:** Pen Yan Jing tablets, pelvic inflammatory disease, Akt, NF-κB

## Abstract

**Purpose:** Pen Yan Jing tablets (PYJ), a Chinese patent medicine, has being used for pelvic inflammatory disease (PID) effectively. This study was designed to explore the underlying mechanisms of PYJ for treating PID.

**Methods:** A rat model of PID was established by mixed bacteria liquid plus mechanical damage. After PYJ treatment, the morphology of uteri and extent of pelvic adhesion were observed. The pathological changes were evaluated by hematoxylin-eosin (HE) staining. The protein expressions of CD68, intercellular cell adhesion molecule-1 (ICAM-1), vascular cell adhesion molecule-1 (VCAM-1), monocyte chemotactic protein-1 (MCP-1) and cyclooxygenase-2 (COX-2) were quantitated by immunohistochemistry. A cell model of lipopolysaccharide (LPS)-activated RAW 264.7 macrophages was performed. The cell proliferation and NO level were measured by CCK-8 and Griess method, respectively. The tumor necrosis factor-α (TNF-α) and interleukin-6 (IL-6) levels were detected by ELISA. The protein kinase B (Akt)/nuclear factor kappa-B (NF-κB) pathway-related protein expressions were assayed by western blot or immunofluorescence.

**Results:** PYJ alleviated pelvic adhesion and inflammatory lesions of uteri in PID rats. PYJ down-regulated protein expressions of ICAM-1, VCAM-1, MCP-1, COX-2, p-Akt, p-IκB kinaseα/β (p-IKKα/β), p-IκBα, p65, and p-p65 in uteri of PID rats. Moreover, PYJ medicated serum inhibited abnormal cell proliferation, NO release, levels of TNF-α and IL-6, nuclear translocation of p65, and protein expressions of p-Akt, p-p65 and p-IκBα in LPS-activated RAW 264.7 macrophages.

**Conclusions**: Taken together, PYJ may alleviates PID through inhibiting Akt/NF-κB pathway.

## Introduction

Pelvic inflammatory disease (PID) is a multiple bacterial infection-induced inflammation disorder in the upper female genital tract, typically involving uteri, fallopian tubes, ovaries and pelvic peritoneum. Left untreated, PID has a high risk of developing severe sequelae, including infertility, ectopic pregnancy and chronic pelvic pain 1. According to a self-reporting USA poll, the incidence of PID has been up to 4.4% 2. Besides, follow-up studies have shown that after 84 months, about 8% of women are infertile, 19.0% have ectopic pregnancies, 42.7% experience chronic pelvic pain, and 21.3% experience recurrent PID 3. Hence, PID is considered as a major threat to women of reproductive age all over the world.

The pathogenesis of PID remains poorly understood. Inflammation is considered to be a key factor contributing to the occurrence and development of PID 1. Persistent inflammation of uteri, fallopian tubes, ovaries and pelvic result in tissue hyperplasia and pelvic adhesion 4. Pro-inflammatory mediators such as adhesion molecules, chemokines and inflammatory cytokines play important roles in regulating the recruitment of leucocytes 5. These mediators trigger an inflammatory cascade through a positive feedback cycle when inflammatory pathways are activated 5. Therefore, reducing pro-inflammatory mediators may represent a therapeutic strategy for treating PID. Additionally, pro-inflammatory macrophage is a major contributor to inflammation and adhesion formation 6-8. Lipopolysaccharide (LPS), a component of the outer membrane of Gram-negative bacteria, induces macrophage infiltration into peritoneal cavity in mice and pro-inflammatory macrophage activation* in vitro* 9. Accordingly, targeting LPS-induced inflammatory response in macrophages may be another potential strategy for ameliorating PID.

PID is commonly treated with antibiotics 10. However, antibiotics have limitations in treating tissue adhesion and hyperplasia and may cause severe adverse reactions 11. Traditional Chinese medicines have been paid more and more attention to treating PID, due to the multitargets, multi-channels, coordination and synergism. Pen Yan Jing tablets (PYJ) is a Chinese patent medicine approved by National Medical Products Administration (approval number: Z20090070). It consists of eight Chinese herbal medicines including *Lonicera japonica* Thunb., *Spatholobus suberectus* Dunn., *Cibotium barometz* (L.) J.Sm., *Taraxacum mongolicum* Hand.-Mazz., *Leonurus japonicus* Houtt., *Plantago asiatica* L., *Paeonia lactiflora* Pall. and *Ligusticum chuanxiong* Hort. at a mass ratio of 25: 25: 25: 12: 12: 12: 6: 6. PYJ and other formulae of Pen Yan Jing have been used clinically to treat PID 12-14. Pen Yan Jing granule, which has the same drug composition with PYJ, exhibits anti-inflammatory and anti-bacterial activities 15. Our previous study shows that PYJ is effective in rats with PID induced by phenolic mucilage 16. However, the underlying anti-PID mechanisms of PYJ have yet to be fully understood.

Here, we investigated the effect of PYJ on PID induced by mixed bacteria liquid plus mechanical damage in rats and LPS-activated RAW 264.7 macrophages. We found that PYJ alleviates PID by inhibiting protein kinase B (Akt)/nuclear factor kappa-B (NF-κB) pathway and thus reducing the production of pro-inflammatory mediators and macrophage activation.

## Materials and Methods

### Chemical Profile Analysis of PYJ by High Performance Liquid Chromatography (HPLC)

PYJ produced by Hutchison Whampoa Baiyunshan Laida Pharmaceutical (Shantou) Co., Ltd. (Guangdong, China; batch No. 190601) was grinded. 1 g of PYJ powder was added into 25 mL of methanol. The mixture was treated with an ultrasonic instrument (200 W, 53 kHZ) for 30 min and then filtered. Standards including protocatechuic acid, catechin, chlorogenic acid, and paeoniflorin (Vicky Biotechnology Co., Ltd., Sichuan, China) were dissolved in methanol. All solutions were filtered through 0.22 μm filter membrane. The HPLC analysis was performed by a Shimadzu HPLC system (LC-20AT, Shimadzu Corporation, Kyoto, Japan) equipped with a Diamonsil-C18 column (4.6 mm × 250 mm, 5 µm; DiKMA Technologies Inc., Beijing, China). The mobile phase was composed of acetonitrile (A) and 0.1% phosphoric acid aqueous solution (B). The gradient elution program was as follows: 0-25 min, 98-90% B; 25-35 min, 90-88% B; 35-45 min, 88-88% B; 45-65 min, 88-85% B. The injection volume was 10 µL and the flow rate was 1.0 mL/min. The column temperature was maintained at 35°C and UV detection wavelength was 220 nm.

### Bacterial Strains and Animals

*Escherichia coli* (CMCC44102), *Staphyloccocus aureus* (ATCC25923) and* β-hemolytic streptococcus* (CMCC32210) were purchased from Beijing Baiou Bowei Biotechnology Co., Ltd (Beijing, China). Female Sprague-Dawley rats, weighing 180-220 g, were provided by Guangdong Medical Laboratory Animal Center (certificate No. SCXK 2018-0002) and were housed at 24.0 ± 0.5°C and a 12 h light/dark cycle lighting schedule in the Biosafety Level 2 Laboratory of the Experimental Animal Center, Guangzhou University of Chinese Medicine (certificate No. SYXK 2018-0001). Rats were allowed free access to food and water. All animal experiments followed the National Institutes of Health Guide for the Care and Use of Laboratory Animals and were approved by the Animal Ethics Committee of Guangzhou University of Chinese Medicine.

### The Establishment of PID Model

The PID model in rats was established as described previously with slight modifications 17, 18. Briefly, rats were anesthetized with an intraperitoneal injection of pentobarbital sodium (36 mg/kg; Merck KGaA, Darmstadt, Germany). A 0.5 cm × 0.5 cm piece of gelatin sponge was inserted into the vagina. Then a 1.0-1.5 cm incision along the midline of the lower abdomen was made to expose the left and right uteri. Both sides were injected with 0.2 mL bacteria mixture (6×10^10^ cfu/mL) of *Escherichia coli*, *Staphylococcus aureus*, and *β-hemolytic streptococcus* (v/v/v, 2:1:1) by an insulin syringe (B. Braun AG, Melsungen, Germany) and the endometrium was scratched by the syringe needle for four times. The uteri of rats in the sham group received an injection of 0.2 mL normal saline without scratch. All surgeries were achieved by the same surgeon. Rats in the normal group did not receive any invasive operation. One week later, treatments in rats were conducted.

### Animals Treatment

64 rats were randomly assigned to 8 groups (*n* = 8) according to body weight: normal group, sham group, model group, three dosages of PYJ groups (388, 775, and 1550 mg/kg/d, p.o.), positive control groups of Fuke Qianjin tablets (FKQJ; 656 mg/kg/d, p.o.; Zhuzhou QianJin Pharmaceutical Co., Ltd., Hunan, China) and dexamethasone tablets (DEX; 0.31 mg/kg/d, p.o.; Guangdong Nanguo Pharmaceutical Co., Ltd., Guangdong, China). The medium dose of PYJ in rats was calculated by the clinical dose of 7.74 g/d in humans based on the body surface area conversion between humans and rats. All tablets were crushed into power and dispersed in 0.5% CMC-Na. The rats in the normal group, sham group, and model group were given 0.5% CMC-Na. Drugs and 0.5% CMC-Na were orally administered once per day for four consecutive weeks.

### Evaluation of Uterine Appearance and Pelvic Adhesion

At the scheduled sampling time point, rats were anesthetized with pentobarbital sodium (36 mg/kg, i.p.). The appearances of uteri were observed. The extent of pelvic adhesion was evaluated by the Philips scoring criteria 19. An investigator blind of treatment arrangement was responsible for the evaluation of pelvic adhesion. Subsequently, the uteri were harvested. The left ones were stored at -80℃, and the right ones were immersed in 10% neutral buffered formalin (Yongjin Biotechnology Co., Ltd., Guangdong, China).

### Histopathologic Analysis

The right uteri fixed in formalin were transferred to processing cassettes, dehydrated through a serial alcohol gradient, embedded in paraffin wax, and sliced into 4-μm-thick sections. Before staining, the sections were dewaxed in xylene and rehydrated through decreasing concentrations of ethanol. Then they were stained with hematoxylin and eosin (HE; Leagene Biotechnology Co., Ltd., Beijing, China). The histopathology of uteri was observed and pictured by a microscope (BX53, Olympus Corporation, Tokyo, Japan).

### Network Pharmacology Analysis Predicted Potential Targets and Signaling Pathways for PYJ Treatment in PID

Encyclopedia of Traditional Chinese Medicine (ETCM) is a database that includes comprehensive and standardized information for the commonly used herbs and formulae of TCM, as well as their ingredients. The ingredients and target genes of PYJ were collected from ETCM (http://www.tcmip.cn/ETCM/). The PID-related target genes were collected from Genecards (http://www.genecards.org), Online Mendelian Inheritance in Man (OMIM; http://omim.org/), and DisGeNET (http://www.disgenet.org/). In the GeneCards database, a higher score value indicates that the target is closely related to the disease. If there are too many targets, targets with a score greater than twice the median are screened as potential targets for PID.

Potential target genes of PYJ therapy for PID were acquired through the Venn 2.1 (https:// bioinfogp.cnb.csic.es/tools/venny/index.html) intersection. The names of the intersecting targets were entered in the STRING 11.0 database (https://string-db.org/) with “Homo sapiens” and “highest confidence (> 0.9)” being selected. The resultant data were then imported into Cytoscape 3.7.2 (www.cytoscape.org/) to construct Protein-Protein Interaction (PPI) network and analysed by the “Network Analyzer” and “MCODE” plug-ins of Cytoscape to obtain the degree of each node and clusters of this network.

The intersecting targets were imported into the Database for Annotation, Visualization and Integrated Discovery (DAVID) version 6.8 (https://david.ncifcrf.gov/) and Gene Ontology (GO) and Kyoto Encyclopedia of Genes and Genomes (KEGG) pathway enrichment analyses were performed. The top 20 significant enrichments (*p* < 0.05) were selected. The enrichment bar graph and enrichment bubble diagram were attained using a website (www.bioinformatics.com.cn).

### Immunohistochemical and Immunofluorescence Analysis

For immunohistochemistry and immunofluorescence staining in uterus paraffin sections, sections were dewaxed and hydrated. The antigen reactivity was recovered in citrate buffer (pH 6.0; Yongjin Biotechnology Co., Ltd., Guangdong, China) or EDTA buffer (pH 9.0; Zhongshan Golden Bridge Biotechnology Co., Ltd., Beijing, China) at 95℃ for 15 min. After washing 3 times with phosphate buffered saline (PBS), the sections were put into 3% hydrogen peroxide solution, incubated in dark for 25 min, and washed 3 times with PBS again. For p65 immunofluorescence staining in cells, cells were washed 3 times with PBS and fixed in 4% paraformaldehyde (Leagene Biotechnology Co., Ltd., Beijing, China) for 15 minutes. Then cells were washed 3 times in PBS and permeabilizated with PBS containing 0.1% Triton X-100 (BioFroxx, Einhausen, German). Tissue slices and cells were blocked in PBS containing 10% goat serum (Zhongshan Golden Bridge Biotechnology Co., Ltd., Beijing, China) for 30 min and then stained in 5% goat serum containing different primary antibodies against CD68 (1:5000; Abcam, Cambridge, UK), intercellular cell adhesion molecule-1 (ICAM-1; 1:100; Huabio, Zhejiang, China), vascular cell adhesion molecule-1 (VCAM-1; 1:500; Abcam, Cambridge, UK), monocyte chemotactic protein-1 (MCP-1; 1:400; Abcam, Cambridge, UK), and cyclooxygenase-2 (COX-2; 1:1600; Abcam, Cambridge, UK), p-IκB kinaseα/β (p-IKKα/β; 1:300; CST, Danvers, MA, USA), and p65 (1:600; CST, Danvers, MA, USA) overnight at 4°C.

For immunohistochemistry staining, the slices were then incubated with second antibodies (1:250; Jackson Immuno Research, West Grove, PA, USA) for 1 h at room temperature. After washing, the tissue sections were covered with diaminobenzidine chromogenic solution until the positive solution became brown. Then the nuclei were stained with haematoxylin for 3 min and rinsed with running water for 10 min. Eventually, a neutral gum seal was applied. For each section, the images of five fields were captured by a microscope at 400× magnification. The average number of CD68 positive staining cells of the five fields was calculated. In terms of ICAM-1, VCAM-1, MCP-1, and COX-2, the ratio of the stained intensity of density (IOD) to the stained area in each image was analysed using Image-Pro Plus 6.0 software (Media Cybernetics, Inc., Rockville, MD, USA). The protein expression, quantified by the average IOD/area ratio of the five fields, was expressed as a fold change relative to the normal group.

For immunofluorescence staining, tissue slices and cells were washed 3 times in PBS and stained with iFluor™ 488 and 594 conjugated goat anti-rabbit IgG goat polyclonal antibodies (Huabio, Zhejiang, China) at a dilution of 1:500 for 1 h at room temperature. tissue slices and cells were washed 3 times in PBS and mounted in DAPI (Dalian Meilun Biotech Co., Ltd., Dalian, China). Images were obtained using a fluorescent microscopy (Guangzhou mshot photoelectric technology Co., Ltd., Guangzhou, China). The mean fluorescence intensity was quantified 5 fields at 400× magnification using ImageJ software (NIH, Bethesda, MD, USA). The protein expression level was expressed as a fold change relative to the normal group.

### Cell Culture

RAW 264.7 cells (Type Culture Collection of the Chinese Academy of Sciences, Shanghai, China), a murine macrophage cell line, were cultured in Dulbecco's modified Eagle's medium (DMEM; Gibco, Carlsbad, CA, USA) supplemented with 10% (v/v) fetal bovine serum (FBS; Biological Industries, Kibbutz Beit Haemek, Israel) and antibiotics (100 units/L penicillin, 100 mg/L streptomycin; Solarbio Science & Technology Co., Ltd., Beijing, China) in a 37°C and 5% CO_2_ incubator.

### Preparation of PYJ Medicated Serum for Cell Treatment

20 rats were randomly distributed into 2 groups (*n* = 10) according to body weight: normal group and PYJ group. The rats in the normal group and PYJ group were administered with 0.5% CMC-Na and PYJ (1550 mg/kg/d, p.o.) once daily for five consecutive days, respectively. 1.5 h after the final administration, all rats were anesthetized with pentobarbital sodium (36 mg/kg, i.p.). Abdominal aorta blood was drawn into vacuum blood collection tubes without anticoagulants and then centrifuged at 3000 rpm for 10 min at room temperature to obtain serum. The serum was incubated at 56℃ for 30 min, sterilized through 0.22 μm filter membrane, and stored at -80℃ before use. Serums from normal and PYJ-treated rats were used as blank serum and PYJ medicated serum, respectively. The blank serum was diluted with serum-free DMEM to a final volume ratio of 10% (v/v). The PYJ medicated serum was diluted with serum-free DMEM and blank serum to final volume ratios of 1%, 5%, or 10% (v/v).

### Cell Proliferation Assay

To investigate the effect of PYJ medicated serum on the proliferation of normal RAW 264.7 cells, cells (2×10^4^ cells per well) were seeded into 96-well plates. After an overnight, cells were incubated with 10% FBS, 10% blank serum, 1%, 5%, or 10% PYJ medicated serum for 24 h. To evaluate the effect of PYJ medicated serum on the proliferation of RAW 264.7 cells stimulated by LPS, cells (3×10^4^ cells per well) were seeded into 96-well plates. After an overnight, the cells were pre-treated with 10% FBS, 10% blank serum, 1%, 5%, or 10% PYJ medicated serum for 24 h and then incubated with LPS (1.0 μg/mL; Sigma-Aldrich Corporation, St. Louis, MO, USA) for 12 h. The images of cells were captured by a microscope after incubation.

For assaying cell proliferation, each well was added with 10% CCK-8 solution (v/v; APE×BIO Technology LLC, Houston, TX, USA) and incubated for 1-4 h. The absorbance at 450 nm was read by a microplate reader (Multiskan GO, Thermo Fisher Scientific Inc., Waltham, MA, USA). The cell proliferation was expressed as a percentage relative to the normal group.

### Determination of NO Release

RAW 264.7 cells were seeded into 6-well plates at a density of 5×10^5^ cells per well and cultured overnight. Thereafter, the cells were pre-treated with 10% FBS, 10% blank serum, 1%, 5%, or 10% PYJ medicated serum for 24 h, followed by LPS incubation (1.0 μg/mL) for 12 h. The supernatants were collected to measure the NO level using a commercial kit (Beyotime Biotechnology Inc., Shanghai, China).

### Determination of Tumor Necrosis Factor (TNF-α) and Interleukin-6 (IL-6) Levels

RAW 264.7 cells were seeded into 96-well plates at a density of 3×10^4^ cells per well and cultured overnight. Afterward, the cells were pre-treated with 10% FBS, 10% blank serum, 1%, 5%, or 10% PYJ medicated serum for 24 h and then incubated with LPS (1.0 μg/mL) for 12 h. The supernatants were collected to determine the levels of TNF-α and IL-6 according to the instructions of commercial kits (Lianke Biotech Co., Ltd., Zhejiang, China).

### Western Blot Analysis

RAW 264.7 cells were pretreated in the same way as the determination of NO release. Then cells were collected and stored at -80℃. The frozen cells and left uteri were lysed in ice-cold RIPA lysis buffer (Cowin Biotech Co., Ltd., Beijing, China) for 30 min and then centrifuged at 12000 g for 10min at 4℃. The supernatants were blended with 5× SDS-PAGE sample buffer (Fude Biological Technology Co., Ltd., Zhengjiang, China), and boiled for 10 min. Proteins were resolved with 10% SDS-PAGE and transferred onto polyvinylidene difluoride (PVDF) membranes (Millipore Corporation, Billerica, MA, USA). The membranes were blocked at 37℃ for 1 h with 5% non-fat milk, cut, and reacted with diluted primary antibodies (1:1000) including inhibitory protein kappa B (IκBα; Abcam, Cambridge, UK), p65, protein kinase B (Akt), p-IKKα/β, p-p65, p-IκBα, p-Akt and GAPDH (CST, Danvers, MA, USA) and IKKα/β (Jiangsu Affinity Biosciences Co., Ltd., Shanghai, China) at 4℃ overnight. The membranes were then incubated with second antibodies (1:5000) at 37℃ for 1 h. The blots were visualized using an ECL reagent (Millipore Corporation, Billerica, MA, USA) and a chemiluminescence imaging system (5200CE, Tanon Science & Technology Co., Ltd., Shanghai, China), and quantified with ImageJ software (NIH, Bethesda, MD, USA).

### Statistical Analysis

Statistical analyses were conducted with SPSS 20.0 software (IBM Corporation, Armonk, NY, USA). The pelvic adhesion score data were expressed as average rank (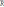) and the statistical analyses were performed by Kruskal-Wallis H test. The quantitative data were expressed as mean ± standard deviation (SD) and the statistical analyses were performed by one-way analysis of variance followed by Least-Significant Difference test (equal variances) or Dunnett's T3 test (unequal variances). A *P* value less than 0.05 was considered statistically significant difference.

## Results

### Chemical Profile of PYJ

**Figure 1:**
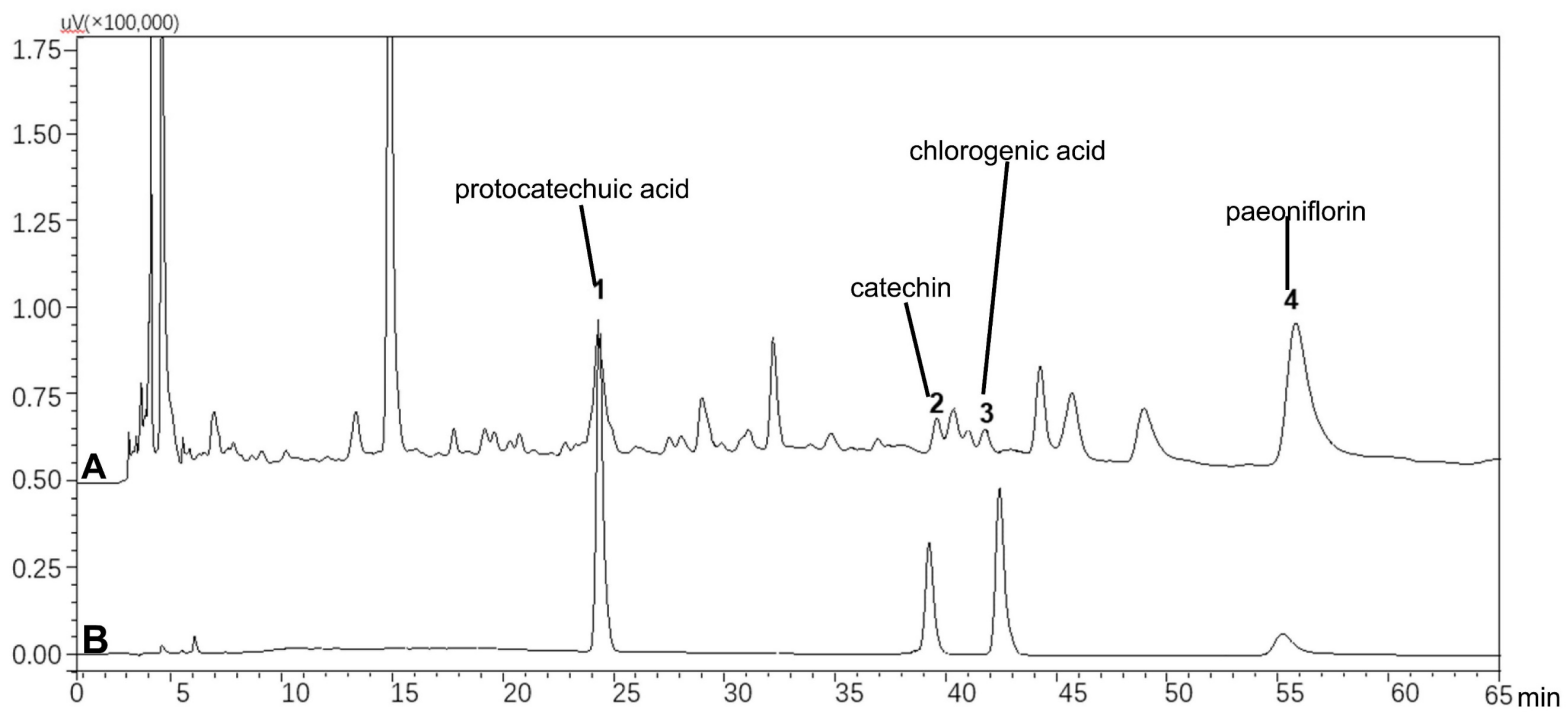
HPLC profiles of (A) PYJ and (B) mixed standards containing protocatechuic acid, catechin, chlorogenic acid, and paeoniflorin.

As shown in Figure 1, PYJ contained protocatechuic acid, catechin, chlorogenic acid, and paeoniflorin.

### PYJ Improved Uterine Appearance and Pelvic Adhesion

**Figure 2:**
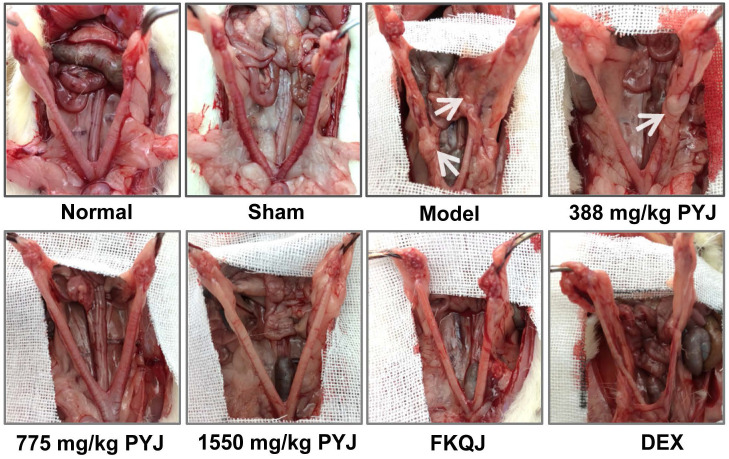
PYJ improved uterine appearance in PID rats. White arrows pointed to pelvic adhesion.

The uteri of normal and sham rats were covered with smooth and pink serosa surface and their shapes were uniform, while the uteri in model rats exhibited uneven thickness, hyperemia, and adhesion to pelvic tissues (Figure 2). These lesions were apparently attenuated by PYJ at doses of 775 and 1550 mg/kg. In addition, as listed in Table 1, compared with the model group, the pelvic adhesion score was remarkably declined in 775 (*P* < 0.05) and 1550 mg/kg (*P* < 0.01) PYJ groups.

### PYJ Ameliorated Histopathologic Changes and Inflammation in Uteri

**Figure 3:**
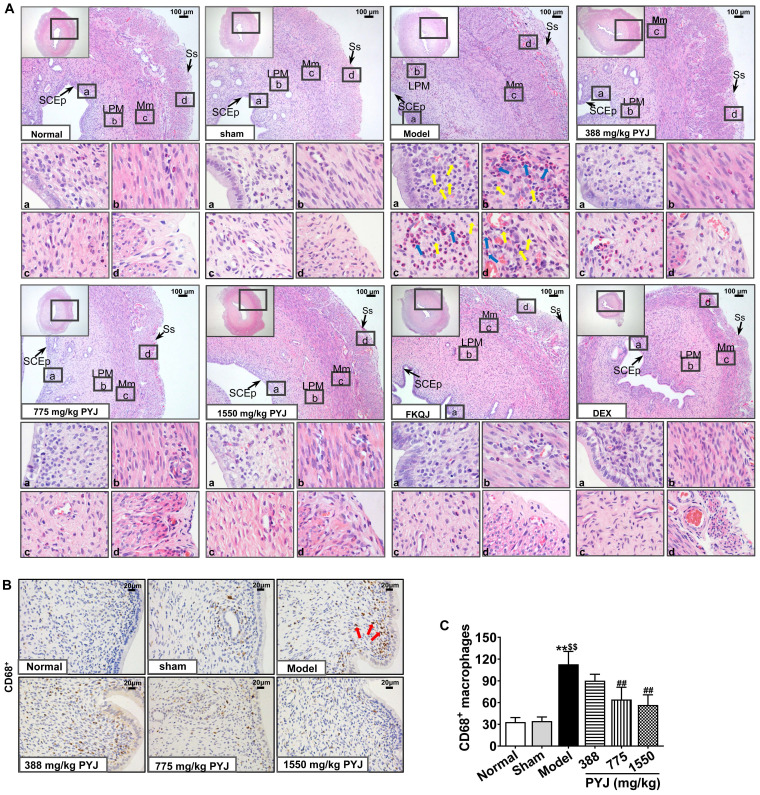
PYJ ameliorated histopathologic changes and inflammation in uteri of PID rats. (A) Representative images of the HE staining of uteri. SCEp, simple columnar epithelium; LPM, lamina propria mucosa; Mm, myometrium; Ss, serosa. Yellow arrows pointed to macrophages. Blue arrows pointed to neutrophils. (B) Representative images of immunohistochemical staining of CD68^+^ macrophages in uteri. Red arrows pointed to CD68^+^ macrophages. (C) The number of CD68^+^ macrophages in uteri. Data were expressed as mean ± SD, *n* = 7-8. ^**^P < 0.01 *vs*. normal group; ^$$^*P* < 0.01 *vs*. sham group; ^##^*P* < 0.01 *vs*. model group.

It was clearly displayed in Figure 3A that the uteri in the normal and sham groups presented complete and clear structures and a few scattered inflammatory cells. Whereas, uterine tissues showed obvious chronic inflammatory pathological changes as follows in the model group. The uterine cavities were narrowed. The number of vessels in lamina propria and myometrium was increased, and these vessels were surrounded by leucocytes, mainly including neutrophils and macrophages. Moreover, the serous layers were thickened and accompanied with enhanced mesothelial cells, fibroblasts, inflammatory cells, collagen fibres, and congested capillaries. In comparison with the model group, leucocytes infiltration in uteri, and collagen fibres and congestion in serous layers were reduced in 775 and 1550 mg/kg PYJ groups (Figure 3A). Furthermore, PYJ at doses of 775 and 1550 mg/kg significantly decreased the number of CD68^+^ macrophages in uteri (*P* < 0.01 *vs.* model group, Figure 3B and C).

### PYJ Reduced the Production of Pro-Inflammatory Mediators in Uteri

**Figure 4:**
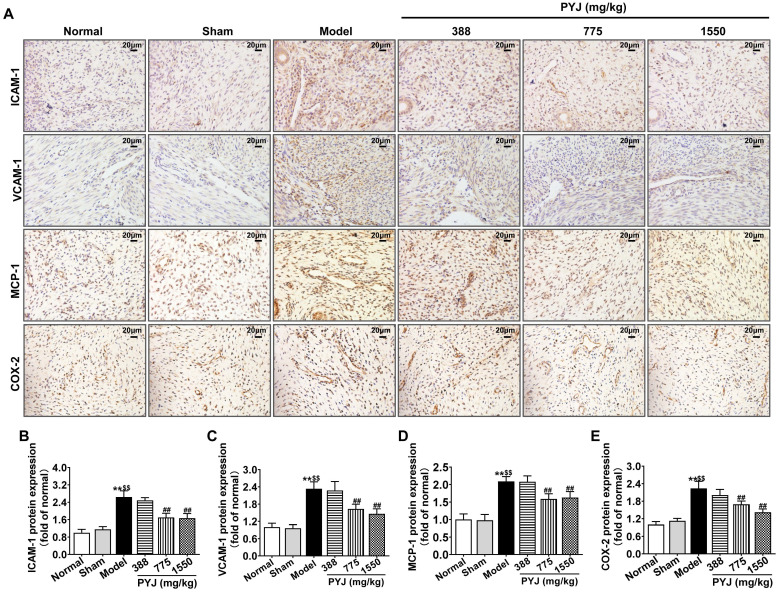
PYJ reduced the production of pro-inflammatory mediators in uteri of PID rats. (A) Representative images of immunohistochemical staining of ICAM-1, VCAM-1, MCP-1, and COX-2 in uteri. (B-E) The protein expressions of ICAM-1, VCAM-1, MCP-1 and COX-2 in uteri evaluated by average IOD/area ratio. Data were expressed as mean ± SD, *n* = 7-8. ^**^P < 0.01 *vs*. normal group; ^$$^*P* < 0.01 *vs*. sham group; ^##^*P* < 0.01 *vs*. model group.

In the model group, ICAM-1, VCAM-1, MCP-1, and COX-2 were highly expressed in uteri (*P <* 0.01 *vs.* normal and sham groups, Figure 4B-E), particularly in leucocytes and/or vascular endothelium (Figure 4A). As compared with the model group, 775 mg/kg PYJ remarkably descended the protein expressions of ICAM-1, VCAM-1, MCP-1, and COX-2 by 35.74%, 30.07%, 24.02%, and 24.41% respectively (*P* < 0.01, Figure 4B-E).

### PYJ Down-Regulated Protein Expressions of Akt/NF-κB Pathway in Uteri

**Figure 5:**
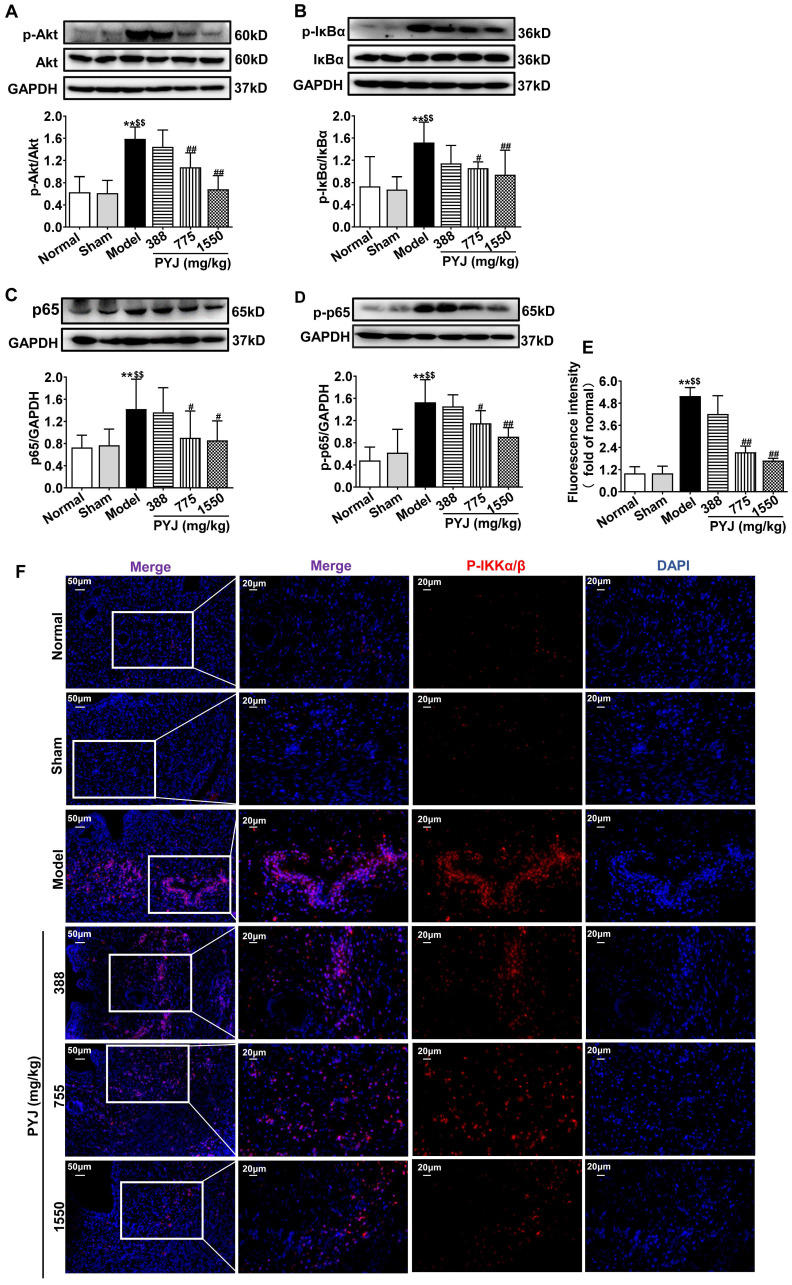
PYJ down-regulated protein expressions related to Akt/NF-κB pathway in uteri of PID rats. Representative western blots and densitometric quantifications of (A) p-Akt, (B) p-IκBα, (C) p65, and (D) p-p65 protein in uteri. (E) Fluorescence intensity p-IKKα/β in uteri. (F) Representative immunofluorescence images showing p-IKKα/β in uteri. Data were expressed as mean ± SD, *n* = 6. ^**^P < 0.01 *vs.* normal group; ^$$^*P* < 0.01 *vs.* sham group; ^#^*P* < 0.05 and ^##^*P* < 0.01 *vs*. model group.

Network pharmacology analysis showed that Akt/NF-κB pathway is one of the potential mechanisms of PYJ in the treatment of PID (Figure S1). As illustrated in Figure 5A-D, the protein expressions of p-Akt, P-IKKα/β, p-IκBα, p65, and p-p65 related to Akt/NF-κB pathway were increased in the model group (*P* < 0.01 *vs.* normal and sham groups), but were restored by 32.01%, 30.44%, 36.49%, and 24.68% respectively in 775 mg/kg PYJ group (*P* < 0.05 or *P* < 0.01 *vs.* model group).

### PYJ Medicated Serum Inhibited Cell Proliferation and Inflammation in Macrophages Stimulated by LPS

**Figure 6:**
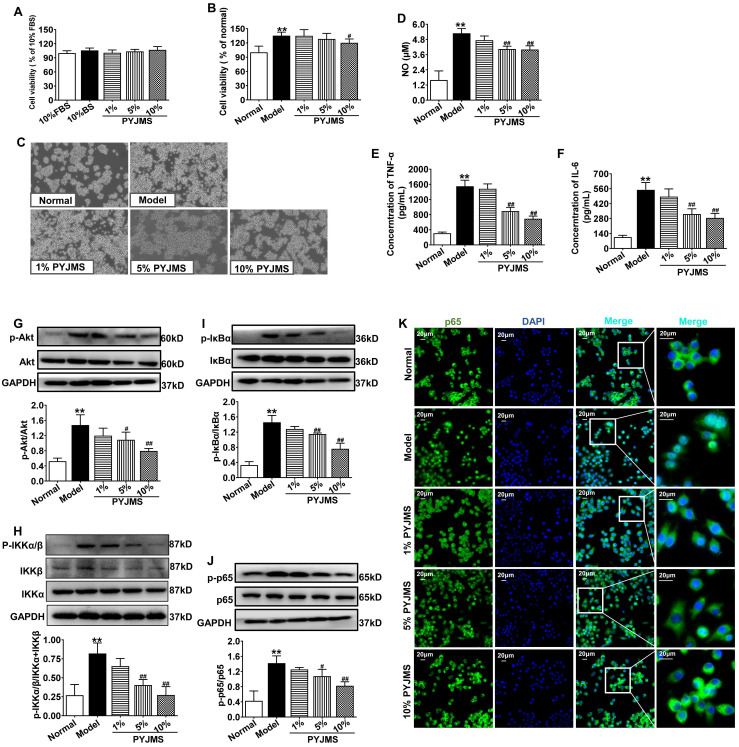
PYJ medicated serum inhibited cell proliferation, inflammation, and protein expressions related to Akt/NF-κB pathway in macrophages stimulated by LPS. (A) PYJ medicated serum showed no effect on cell viability of normal RAW 264.7 cells. (B) PYJ medicated serum inhibited cell proliferation of RAW 264.7 cells stimulated by LPS. (C) Representative images of RAW 264.7 cells. (D) The NO release and levels of (E) TNF-α and (F) IL-6 in RAW 264.7 cells. Representative western blots and densitometric quantifications of (G) p-Akt, (H) p-IKKα/β, (I) p-IκBα and (J) p-p65 in RAW 264.7 cells. (F) Representative immunofluorescence images showing p65 distribution in RAW 264.7 cells. Data were expressed as mean ± SD, *n* = 6 for A, B, E and F, *n* = 3 for D and G-J. ^**^P < 0.01 *vs.* normal group; ^#^*P* < 0.05 and ^##^*P* < 0.01 *vs*. model group. FBS, fetal bovine serum; BS: blank serum; PYJMS, PYJ medicated serum.

There was no statistical difference in cell viability of normal RAW 264.7 cells receiving different treatments (Figure 6A), which suggests that 1%-10% PYJ medicated serum had no cytotoxicity. When normal RAW 264.7 cells were stimulated by LPS, a significant increase in cell proliferation was observed (*P* < 0.01 *vs.* normal group, Figure 6B and C). However, the cell proliferation can be inhibited by treatment with 10% PYJ medicated serum by 11.09% (*P* < 0.05 *vs.* model group). In addition, the levels of NO, TNF-α, and IL-6 were dramatically increased in LPS-stimulated RAW 264.7 cells (*P* < 0.01 *vs.* normal group, Figure 6D-F), while 10% PYJ medicated serum reversed these by 23.56%, 55.39%, and 47.77%, respectively (*P* < 0.01 *vs.* model group).

### PYJ Medicated Serum Down-Regulated Protein Expressions of Akt/NF-κB pathway in Macrophages Stimulated by LPS

As shown in Figure 6G-I, the protein expressions of p-Akt, p-IKKα/β, p-IκBα, and p-p65 in RAW 264.7 cells were increased after LPS stimulation (*P* < 0.01 *vs.* normal group), while pre-treatment with 10% PYJ medicated serum significantly supressed these proteins by 46.16%, 50.56%, 47.92%, and 42.12%, respectively (*P* < 0.01 *vs.* model group). Furthermore, LPS remarkably promoted NF-κB/p65 nucleus translocation. However, 5%-10% PYJ medicated serum also prevented the nucleus translocation of NF-κB/p65.

## Discussion

PID is considered as a major threat to women at reproductive age all over the world. Delayed diagnosis causes inflammatory sequelae, including infertility, ectopic pregnancy, and chronic pelvic pain. PYJ has been used for PID in clinic 12-14. Our previous study demonstrates that PYJ is effective for PID induced by phenolic mucilage in rats 16. However, the underlying anti-PID mechanisms of PYJ are still unknown. In the present study, we found that PYJ alleviates PID by inhibiting the Akt/NF-κB pathway and thus reducing the production of pro-inflammatory mediators and macrophage activation.

In this study, a rat model of PID induced by injecting a mixture of bacterial infections plus mechanical damage in the uterus was utilized to investigate the underlying anti-PID mechanisms of PYJ, because this model resembled the pathogenesis of PID in humans closely. The apparent changes like severe pelvic adhesion and abnormal morphology were observed in uteri (Table 1 and Figure 2). Furthermore, uterine pathology also showed chronic inflammation such as leucocytes infiltration, tissue hyperplasia, and increased collagen fibres (Figure 3A). These results suggest that the PID model is established successfully. As expected, PYJ improved uterine morphology, pelvic adhesion, and uterine inflammation of PID rats. What's more, with the activities of anti-inflammation, reducing collagen deposition, and inhibiting fibrocyte DNA synthesis and granulomas proliferation, DEX is often used as a positive control in anti-PID drug studies and is reported to be effective for phenolic mucilage-induced PID 16,20. However, in our study, DEX not only had no protective effect on PID (Table 1 and Figure 2 and 3A), but also resulted in the death of three rats. The reason may be that we applied a different PID modelling method that is mixed bacteria liquid plus mechanical damage. The immunosuppressive effect of DEX further worsens the bacterial infections in PID rats.

Macrophages play a distinctive role in chronic inflammation and adhesion formation 6,8. CD68 is a heavily glycosylated protein and has been widely employed as a macrophage marker 8. In this study, PYJ reduced the number of CD68^+^ macrophages in uteri of PID rats (Figure 3B and C). In addition, PYJ medicated serum also inhibited the abnormal proliferation of macrophages stimulated by LPS* in vitro* (Figure 6B and C). These results suggest that inhibiting excessive activation of macrophages may contribute to the anti-PID effects of PYJ.

Adhesion molecules and chemokines are crucial to the recruitment of inflammatory cells 21. ICAM-1 and VCAM-1, as essential adhesion molecules, promote the migration of leucocytes from blood to inflammation tissues 21. Moreover, ICAM-1 is important for the development of intestinal adhesion 22. In a mouse model of ovalbumin-induced lung inflammation, VCAM-1 antibody inhibits the recruitment of macrophages, neutrophils, and eosinophils 23. MCP-1, also known as chemokine (C-C motif) ligand 2 (CCL2), is a ligand on the surface of monocytes and has a specific chemotactic activation effect on monocytes. MCP-1 is closely related with diseases characterized by monocyte infiltration such as rheumatoid arthritis 24. What's more, COX-2 plays an important role in regulating inflammation and angiogenesis in the development of postoperative adhesion 25. Therefore, ICAM-1, VCAM-1, MCP-1, and COX-2 are key pro-inflammatory mediators and contribute to leucocytes recruitment and adhesion formation. Our results showed that PYJ remarkably descended protein expressions of ICAM-1, VCAM-1, MCP-1, and COX-2 in uteri of PID rats (Figure 5), especially in the sites of inflammatory cells infiltration, suggesting PYJ attenuates chronic inflammation and pelvic adhesion of PID by suppressing these pro-inflammatory mediators.

In addition, pro-inflammatory cytokines also lead to chronic inflammation by positive feedback loops 26. Both IL-6 and TNF-α induce the production of ICAM-1, VCAM-1, MCP-1, and COX-2, amplifying leucocyte recruitment 27-29. Besides, NO is produced from nitric oxide enzyme catalysed by L-arginine and participated in the release of pro-inflammatory cytokines in macrophages 30. LPS triggers a cytokine storm characterized by myriad pro-inflammatory mediators in macrophages 31. We found that PYJ medicated serum suppressed LPS-induced secretion of NO, TNF-α, and IL-6 in macrophages (Figure 6D-F), which explains the inhibitory effects of PYJ on macrophage inflammation and pelvic adhesion in PID.

It has been demonstrated that NF-κB is an important transcription factor of pro-inflammatory mediators 32. Akt, also known as protein kinase B, participates in inflammatory mediator production, chemotaxis, migration, and survival of macrophages by regulating NF-κB 33,34. Physiologically, NF-κB, a heterodimer commonly composed of p50 and p65, forms a complex with IκBα and locates in cytoplasm 32. After pattern recognition receptors being activated by pathogens, Akt is phosphorylated and thus activated. The phosphorylated Akt phosphorylates IKK and thereby phosphorylates IκBα 35. Then phosphorylated IκBα dissociates from the complex of NF-κB, and finally degrade, while NF-κB is thus phosphorylated and translocates into nuclear. NF-κB was reported to promoting the gene transcriptions of IL-6, TNF-α, ICAM-1, VCAM-1, MCP-1, and COX-2 36. As predicted by network pharmacology, Akt and NF-κB were great potential targets of PYJ against PID (Figure S1). Furthermore, the phosphorylation of p-p65, p-IκBα, p-IKKα/β, and p-Akt were up-regulated in both uteri of PID rats and LPS-stimulated RAW 264.7 cells (Figure 6 and 6G-I), indicating Akt/NF-κB pathway is activated. Nevertheless, PYJ dramatically attenuated the upregulated phosphorylation of protein related to Akt/NF-κB pathway. Taken collectively, it can be inferred that PYJ attenuates the activation of Akt/NF-κB pathway, therefore inhibiting infiltration of macrophages and production of pro-inflammatory mediators in PID.

In addition, the IκBα-independent termination of NF-κB transcriptional activity is dependent on the ubiquitination and proteasomal degradation of p65 37. A number of ubiquitin ligases for NF-κB have been identified, such as suppressor of cytokine signaling 1, PDZ, and LIM Domain 2 38. In the present study, p65 protein expression was significantly up-regulated in uteri of PID rats and was attenuated by PYJ (Figure 5C). The inhibitory effect of PYJ on p65 may be attributed to promoting ubiquitination and proteasomal degradation of p65.

PYJ contained four bioactive components including protocatechuic acid, catechin, chlorogenic acid, and paeoniflorin (Figure 1). These components attenuate inflammatory pain and neuroinflammation by inhibiting Akt and NF-κB 39-42, which may be responsible for the protective effect of PYJ on PID.

## Conclusion

**Figure 7:**
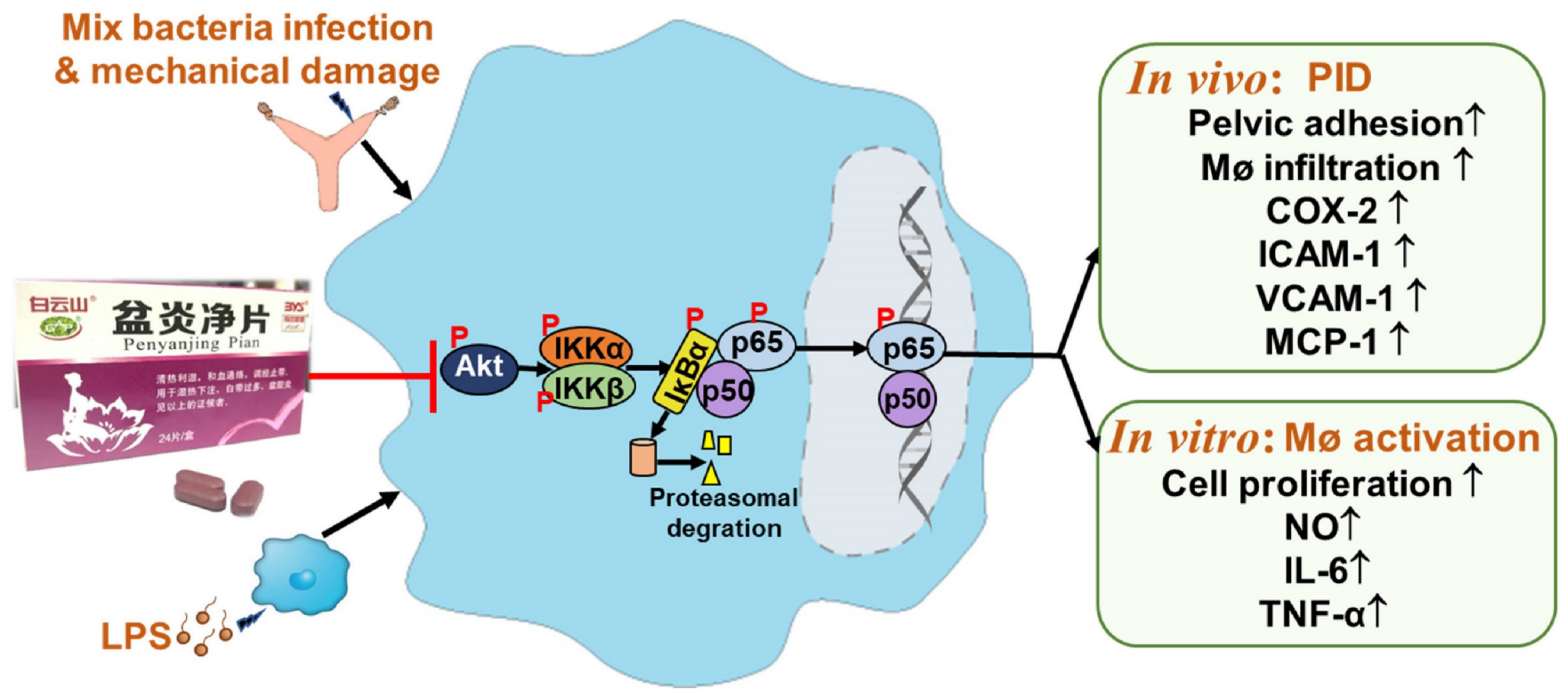
PYJ alleviates PID via inhibiting Akt/NF-κB pathway.

In summary, PYJ alleviates PID by suppressing the production of pro-inflammatory mediators and macrophage activation. The anti-PID mechanism of PYJ may be related to inhibiting Akt/NF-κB pathway (Figure 7).

## Supplementary Material

Figure S1 Network pharmacology analysis for PYJ treatment in PID.

## Figures and Tables

**Table 1:** PYJ alleviated pelvic adhesion in PID rats (*f*, 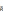, *n* = 10-16)

Group	Uterus (*n*)	Pelvic adhesion score					
		0	Ⅰ	Ⅱ	Ⅲ	Ⅳ	
Normal	16	16	0	0	0	0	28.00
Sham	16	14	2	0	0	0	33.38
Model	16	2	5	1	5	3	88.78^**$$^
388 mg/kg PYJ	16	3	5	5	2	1	78.00
775 mg/kg PYJ	16	5	8	3	0	0	59.29^#^
1550 mg/kg PYJ	16	8	5	3	0	0	54.28^##^
FKQJ	16	5	6	5	0	0	65.53^#^
DEX	10	2	1	3	4	0	86.25

**P < 0.01 *vs*. normal group; ^$$^*P* < 0.01 *vs*. sham group; ^#^*P* < 0.05 and ^##^*P* < 0.01 *vs*. model group.

## Abbreviations

Akt: protein kinase B
COX-2: cyclooxygenase-2
CCL2: chemokine (C-C motif) ligand 2
DAVID: database for annotation, visualization and integrated discovery
DEX: dexamethasone tablets
DMEM: Dulbecco's modified Eagle's medium
ETCM: encyclopedia of traditional Chinese medicine
FBS: fetal bovine serum
FKQJ: Fuke Qianjin tablets
GO: gene ontology
HE: hematoxylin-eosin
ICAM-1: intercellular cell adhesion molecule-1
HPLC: high performance liquid chromatography
IκBα: inhibitory protein kappa B
IL-1β: interleukin-1β
IL-6: interleukin-6
IOD: intensity of density
KEGG: kyoto encyclopedia of genes and genomes
LPS: lipopolysaccharide
MCP-1: monocyte chemotactic protein-1
NF-κB: nuclear factor-kappa B
OMIM: online mendelian inheritance in man
PBS: phosphate buffered saline
PID: pelvic inflammatory disease
PIK3R1: phosphatidylinositol 3-kinase regulatory subunit alpha
PPI: protein-protein interaction
PTGS1: prostaglandin-endoperoxide synthase 1
PVDF: polyvinylidene difluoride
PYJ: Pen Yan Jing tablets
IKKα/β: IκB kinase α/β
SD: standard deviation
TNF-α: tumor necrosis factor-α
TLR4: Toll-like receptor 4
VCAM-1: vascular cell adhesion molecule-1
