# Supplementary material for: Pen Yan Jing Tablets Alleviates Pelvic Inflammatory Disease by Inhibiting Akt/NF-κB Pathway
Source: Int J Med Sci. 2023 Sep 4;20(11):1386–98. doi: 10.7150/ijms.87433 (PMC10542183; doi:10.7150/ijms.87433)
Supplement: Supplementary file 1 — Figure S1 Network pharmacology analysis for PYJ treatment in PID. [file ijmsv20p1386s1.pdf]

Supplementary material

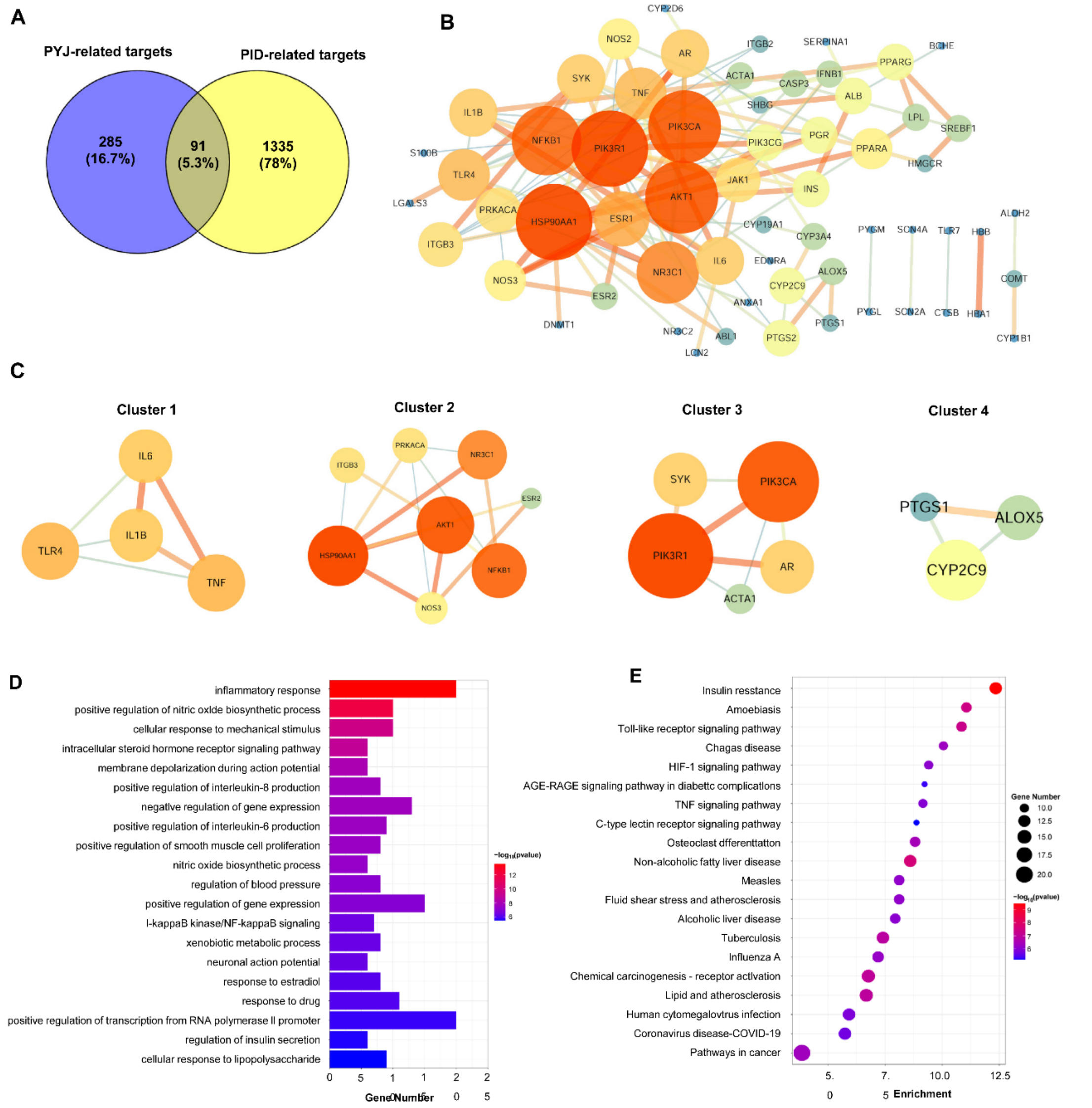

Figure S1 Network pharmacology analysis predicted potential targets and signaling pathways for PYJ treatment in PID. (A) The Venn diagram of overlap targets between 376 candidate PYJ-related targets and 1426 PID-related targets. (B) The PPI network diagram of PYJ targets against PID in the STRING database. (C) Four clusters of PPI network. The circular nodes represent the interacting proteins that directly or indirectly interact with each other. The node size represents the importance of proteins and the thickness of the line represents the closeness of the relationship between proteins. The top 20 enriched (D) GO terms for biological process and (E) KEGG pathways of 91 targets of PYJ in PID.
